# Prediction of solubility on recombinant expression of *Plasmodium falciparum *erythrocyte membrane protein 1 domains in *Escherichia coli*

**DOI:** 10.1186/1475-2875-5-52

**Published:** 2006-06-25

**Authors:** Sanjay Ahuja, Satpal Ahuja, Qijun Chen, Mats Wahlgren

**Affiliations:** 1 Department of Microbiology, Tumor and Cell Biology (MTC), Karolinska Institutet, P. O. Box 280, Stockholm, S-17177, Sweden; 2 Wallenberg Retina Centre, Department of Ophthalmology, Lund University, BMC-B13, Klinikgatan 26, Lund, 221 84, Sweden

## Abstract

**Background:**

Cellular interactions elicited by *Plasmodium falciparum *erythrocyte membrane protein antigen 1 (PfEMP1) are brought about by multiple DBL (Duffy binding like), CIDR (cysteine-rich interdomain region) and C2 domain types. Elucidation of the functional and structural characteristics of these domains is contingent on the abundant availability of recombinant protein in a soluble form. A priori prediction of PfEMP1 domains of the 3D7 genome strain, most likely to be expressed in the soluble form in *Escherichia coli *was computed and proven experimentally.

**Methods:**

A computational analysis correlating sequence-dependent features to likelihood for expression in soluble form was computed and predictions were validated by the colony filtration blot method for rapid identification of soluble protein expression in *E. coli*.

**Results:**

Solubility predictions for all constituent PfEMP1 domains in the decreasing order of their probability to be expressed in a soluble form (% mean solubility) are as follows: ATS (56.7%) > CIDR1α (46.8%) > CIDR2β (42.9%) > DBL2-4γ (31.7%) > DBL2β + C2 (30.6%) > DBL1α (24.9%) > DBL2-7ε (23.1%) > DBL2-5δ (14.8%). The length of the domains does not correlate to their probability for successful expression in the soluble form. Immunoblot analysis probing for soluble protein confirmed the differential in solubility predictions.

**Conclusion:**

The acidic terminal segment (ATS) and CIDR α/β domain types are suitable for recombinant expression in *E. coli *while all DBL subtypes (α, β, γ, δ, ε) are a poor choice for obtaining soluble protein on recombinant expression in *E. coli*. This study has relevance for researchers pursuing functional and structural studies on PfEMP1 domains.

## Background

The recent completion of sequencing of the genome of *Plasmodium falciparum *has stimulated studies on new proteins and biochemical systems that need further experimental validation. This influx of potentially useful vaccine and drug candidates against malaria has added to the backlog of targets currently under validation. The bottleneck, imposed by the need to have soluble proteins for functional studies, has been considerably narrowed by the unpredictability associated with recombinant expression in host systems. Protein transition, from a naturally occurring low concentration *in vivo*, to unnaturally higher concentrations in expression hosts often results in poor solubility, aggregation and inclusion body formation [1]. The success of most, if not all functional and structural studies, is contingent on the abundant availability of soluble proteins.

Heterologous expression of malarial proteins is performed primarily in *Escherichia coli*, although yeast and insect cells are becoming increasingly popular, particularly for proteins that are predicted to undergo post-translational processing. In general, *E. coli, as *an expression host has advantages in terms of procedural simplicity, well known *E. coli *genetics, availability of compatible molecular tools, higher yields per unit biomass and thereby low costs sustained. The fact that over expression in *E. coli *is often associated with accumulation of recombinant protein as insoluble aggregates has not precluded its usage as the expression system of primary choice. A wide arsenal of innovative strategies has been employed to increase the yield of soluble protein(s). These include simultaneous over expression of molecular chaperones, remedying codon bias with supplementation of rare tRNAs, mRNA stabilisation strategies, use of strong promoters with tight control of background expression in protease defecient *E. coli *strains and optimization of culture media against limiting factors and products [2]. These strategies although useful, however, cannot be harnessed in a high throughput manner. Malarial genes exhibit an A+T composition approaching 80% and show frequent occurrence of long clusters of rare *E. coli *codons. Consequently, premature translational termination, frameshift events and mistranslational amino acid substitutions arising from codon bias further complicate recombinant expression of malarial proteins in *E. coli*. Recombinant expression of malarial genes in *E. coli *is thus confined to trial and error.

In this setting, prior knowledge of genes or sequences that either do or do not lend to soluble expression in *E. coli *is thus of particular relevance. Mutagenesis studies provide indirect evidence to the effect of sequence on solubility and propensity of a polypeptide chain to aggregate into inclusion bodies. Alteration in the primary sequence of a protein by mutational replacement of non-polar amino acid residues with charged ones such as lysine has been shown to enhance intrinsic solubility of recombinant proteins [3]. Specific mutations that alter the overall charge of the peptide molecule without altering its overall hydrophobic nature or structure have been shown to influence aggregation [4]. Additionally, sequence related factors such as hydrophobicity, hydrophilic-hydrophobic patterning, charge, high β-sheet and low β-turn propensities have all been implicated in the aggregation of proteins [5]. A pragmatic application that exploits the correlation between sequence and solubility is evident in the screening of expression libraries. Fusion of mutants of the target protein to a reporter with a selectable function e.g green fluorescence protein allows for easy selection of soluble mutants for functional studies [6].

To date, characterization of many of the well-studied proteins has been possible, partly due to their higher solubility on recombinant expression. On the other hand, there are many other proteins that have not been amenable for biophysical studies because of a tendency to aggregate. Attempts have been made to compare these two groups of proteins on the basis of physicochemical parameters that are determined by amino acid composition and are hypothesized to be related to *in vivo *solubility. Based on an in-depth analysis of the physiochemical parameters of 81 proteins for which experimental data was available, protein charge and relative number of turns were the two parameters found to correlate strongly with the formation of inclusion bodies [7].

A strategy often employed to characterise complex proteins is to study them at the level of individual domains. This strategy has been effectively employed to study *P. falciparum *erythrocyte membrane protein 1 (PfEMP1). Each PfEMP1 molecule contains 4–7 extra cellular domains anchored to the erythrocyte surface through a transmembrane region and an acidic terminal segment (ATS) [8]. These domains, namely DBLα-ε (Duffy binding like domains), CIDRα-γ (cysteine-rich interdomain region) and C2 are implicated in binding to endothelial receptors, uninfected erythrocytes and platelets and are thus subject to structure – function investigations [9-11]. The latter is largely dependent on the availability of soluble protein expressed recombinantly.

An analysis conducted by Wilkinson and Harrison [7] was applied to all the PfEMP1 domains of the 3D7 genome parasite. Solubility predictions for individual domains were calculated and comparison across domain groups was conducted. Correlations between the domain length (number of amino acid residues) per domain and propensity for expression in soluble form were determined. Further, solubility predictions for a limited set of PfEMP1 domains were confirmed by recombinant expression in *E. coli*.

## Methods

### Dataset

DNA and protein sequences corresponding to PfEMP1 from *P. falciparum *3D7 genome strain were retrieved from the database maintained by the National Center for Biotechnology Information (PlasmoDB). PfEMP1 sequences were aligned and each sequence was further divided manually into constituent domains. The domain boundaries were defined according to previously published criteria [8].

### Solubility predictions

A statistical model for prediction of solubility on expression in *E. coli*, as defined by Wilkinson and Harrison [7], was used for this study. This model has been primed on 81 proteins for which expression results are available. Discriminant analysis was used to compare these proteins according to six composition related parameters *viz*. – charge average, turn forming residue fraction, cysteine fraction, proline fraction, hydrophilicity and total number of amino acid residues. The relative number of turn forming residues (asparagine, glycine, proline and serine) and absolute charge per residue (fraction of positively and negatively charged amino acids), were found to correlate strongly with inclusion body formation. A composite parameter (CV-canonical variable) dependent on the contribution of each of the individual amino acid was derived and is as follows:

CV = 15.43 {(N + G + P + S)/n} - 29.56 {[(R + K) - (D + E)/n] - 0.03}

where N, G, P, S, R, K, D, E are the absolute numbers of asparagine, glycine, proline, serine, arginine, lysine, aspartic acid and glutamic acid residues, respectively, and n is the total number of residues in the whole sequence. A threshold discriminate CV' = 1.71 [12] was introduced to distinguish soluble proteins from insoluble ones. A protein is predicted to be soluble, if the difference between CV and CV' is negative. On the contrary, a CV-CV' difference larger than zero, predicts the protein to be insoluble. Further a probability of solubility was calculated from the following equation:

P = 0.4934 + 0.276 (CV-CV') - 0.0392(CV-CV')^2 ^[12]

Using the percentage probabilities to classify proteins as soluble or insoluble, discriminant analysis successfully classifies proteins as being soluble or insoluble with an overall accuracy of 88% [7].

For the PfEMP1 domain dataset, the CV-CV' values, probabilities for soluble expression in percentage, relative number of turn forming residues, charge per residue and length of protein sequence were compared. Additionally mean solubility propensities along with the lower and upper quartiles for each domain group were compared.

### Expression constructs

Two sequences of similar length but belonging to different domain types, namely DBL1α (PF13 0003/3D7) and CIDR1α (PF08 0106/3D7) were PCR amplified with the following primers:

5'-ATC GAG CTC TCA CTT ACA TAC AAA TTT CAT ACT AAT-3' and 5'-GAT CTC GAG GTA TTT TTT CTT TTG TTT TTA AAA TTC TT-3' for DBL1α PF13 0003, 5'-ATC GAG CTC TGT GAA AAA GTT GAC GAC GAA GAA-3' and 5'-GAT CTC GAG GGT GGC GTC TTT TAG TTC CTC T-3' for CIDR1α PF08 0106.

The amplified PCR fragments were purified using a Qiagen Purification Kit (Qiagen, CA, USA). The fragments were subsequently digested with compatible restriction enzymes (New England Biolabs, MA, USA) and ligated with Fast-Link TM DNA Ligation Kit (Epicentre Technologies, WI, USA) to double-digested and gel-purified pQE-Tri-System His-Strep 2 vector (Qiagen, CA, USA). The recombinant plasmids were transformed into TOPO competent cells (Invitrogen, CA, USA) and positive recombinant clones were confirmed with PCR and restriction enzyme digestion. All constructs were verified by DNA sequencing in an ABI377 automated sequencer with a Big Dye terminator v3.1 cycle sequencing kit (Appied Biosystems, CA, USA). Another plasmid construct for the expression of ATS (FCR3S1.2) was generated as described earlier [9]. SG10009 chemically competent *E. coli *cells (Qiagen, CA, USA) were transformed with the histidine (His) and glutathione S-transferase (GST) expression constructs.

### Expression in *E. coli*

Experimental validation of solubility predictions was conducted by employing a new method, namely colony filtration blot for rapid identification of soluble protein expression in *E. coli *[13]. This method is based on the separation of soluble protein from inclusion bodies by a filtration step at the colony level and has previously been used to screen expression constructs from a deletion mutagenesis library with 84% specificity for discrimination between soluble and insoluble expression products in the Rosetta™ (DE3) pLysS *E. coli *strain. In short, cells harbouring the expression construct were plated and grown on an LB plate. After overnight growth, a 0.45 μm Durapore filter membrane (Millipore, MA, USA) was placed on top of the colonies. The membrane was peeled off and placed carefully, with the colonies facing upwards, on a LB plate containing 0.2 mM IPTG. Recombinant protein expression in the colonies "on the membrane" was induced for 6 hours at room temperature. The filter membrane was subsequently peeled off and placed on top of a nitrocellulose filter and a Whatman paper, both soaked in lysis buffer [20 mM Tris pH 8.00, 100 mM NaCl, 0.1 mg/ml Lysozyme, 0.75 mg/ml DNAse I, 1/2 complete EDTA-free protease inhibitor cocktail tablet/10 ml (Roche, Basel, Switzerland)]. The "filter-sandwich" was incubated at room temperature for 30 min and then freeze-thawed thrice for 10 min at -80°C and 37°C respectively. The nitrocellulose membrane was subsequently removed from the sandwich and blocked with 1% BSA in TBST (20 mM Tris, pH 7.5, 500 mM NaCl, 0.05% Tween 20) for 1 hour. The membrane was washed 3 × 10 min in TBST and incubated for another hour with a mouse monoclonal Penta-His antibody (Qiagen, Hilden, Germany) or anti-GST mouse monoclonal antibody (Sigma, USA) in1: 1000 dilution in TBST. After incubation with the primary antibody, the membrane was washed 3 × 10 min in TBST. Following the washes, an alkaline phosphatase-labelled anti-mouse polyclonal secondary antibody (Dako, Denmark) was added to the membrane at a 1:1000 dilution in TBST. Reactive protein spots were visualised by incubation with the enzyme substrate nitroblue tetrazolium-5-bromo-4-chloro-3-indoyl phosphate (Sigma, USA).

## Results and discussion

**Figure 1:**
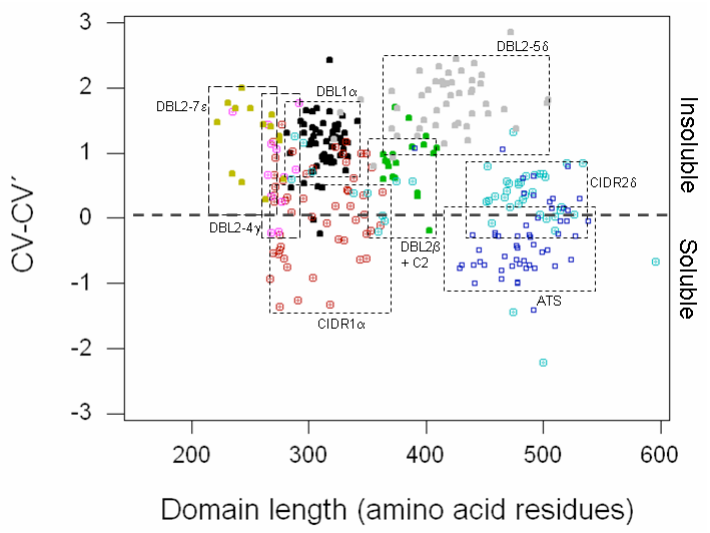
**Distribution of CV-CV' values for all domains harboured on PfEMP1 molecules of the 3D7 genome parasite**. CV-CV' values computed for all PfEMP1 domains were plotted against domain length. Negative CV-CV' values predict the domain to be soluble, while positive values predict the domain to be insoluble on expression in *E. coli*. Dashed boxes enclose all domains belonging to a particular domain type. ATS domains are plotted as squares (dark blue), CIDR domains as circles with a central dot (CIDRα – red, CIDR2β – cyan) and DBL domains as solid circles (DBL1α – grey, DBL2β + C2 – green, DBL2-4γ – magenta, DBL2-5δ – grey and DBL2-7ε – yellow).

**Figure 2:**
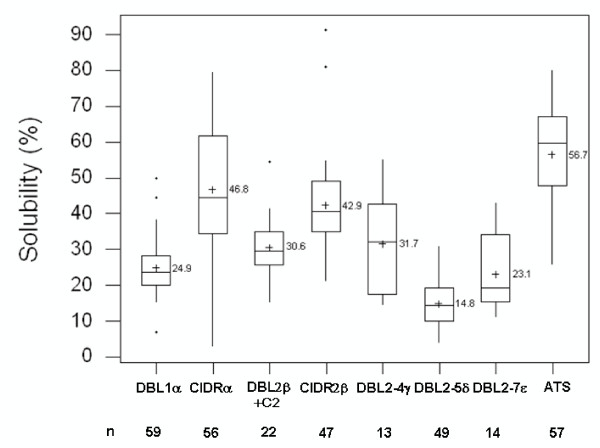
**Box Plot summarizing predictions for obtaining soluble protein on recombinant expression of all constituent domain types on PfEMP1 molecules**. The boxes illustrate the first and third quartiles with whiskers extending over the interquartile range of the first and third quartiles. A horizontal line placed across the width of the box illustrates the median. Outliers are indicated by asterisk (*) and means by a plus sign (+). The numerical mean solubility for each domain is indicated adjacent to the latter. The frequency (n) of each domain in the dataset is indicated below the respective domains.

Recombinant expression of different PfEMP1 domains in *E. coli *often presents the simplest approach for obtaining protein for receptor ligand studies. Probability of solubility analysis of these domains according to Wilkinson and Harrison [7], however, illustrates that the domains exhibit considerable heterogeneity as regards to their propensity for expression as soluble proteins. From amongst all PfEMP1 domain types, the ATS domains stand out as the ones most suitable for expression in *E. coli*. Seventy one percent of the analysed ATS sequences are predicted to exhibit expression in soluble form (Figures 1 and 2). On the other extreme, almost all of the DBL2-5δ sequences are predicted to exhibit low solubility of the recombinant protein expressed in *E. coli*. Out of the 57 ATS domains analysed, the mean solubility for this domain types is predicted to be 56.7% (range: 25.7% – 80.3%). In comparison, the average solubility for the DBL2-5δ domain type is predicted to be 14.8% (range: 3.8% – 31.1%) and is thereby unsuitable for expression in *E. coli*. The DBL1α and CIDR1α domain types, which in tandem comprise PfEMP1 "head-structure", however, exhibit different propensities for expression in a soluble form in *E. coli*. While a significant proportion of CIDR1α domains were predicted to result in soluble expression, the opposite was true for the DBL1α domains. The mean solubility for CIDR1α domains, while being only marginally lower than that of ATS domains, is approximately twice that of DBL1α domains (46.8% vs. 24.9%). The CIDR2β domain, a domain that exhibits significant internal conservation and is closely related to the CIDR1α domain, was predicted to exhibit 42.4% probability for expression in soluble form in *E. coli*. The DBL2β + C2 domain, a tandem domain implicated in intercellular adhesion molecule 1 (ICAM-1) binding, was predicted to be fairly insoluble (30.6% mean probability of solubility) on recombinant expression in *E. coli*. The DBL2-4γ and DBL2-7ε domains – domain types encountered infrequently on PfEMP1 molecules, were also predicted to give low soluble expression in *E. coli *(mean solubility probabilities of 31.6% and 23.1% respectively). Taken together a gradient in probabilities for soluble expression for PfEMP1 domain types was observed. All PfEMP1 domains could thus be ordered in decreasing order of their probability for soluble expression as follows:

ATS > CIDR1α > CIDR2β > DBL2-4γ > DBL2β + C2 > DBL1α > DBL2-7ε > DBL2-5δ

DBL domains in general are poor choices for obtaining recombinant protein in soluble form on expression in *E. coli*. On the contrary ATS and CIDR domains are fairly good choices for obtaining soluble protein on recombinant expression in *E. coli*.

The solubility predictions computed according to the model proposed by Wilkinson and Harrison [7] were confirmed experimentally in a small expression series in *E. coli*. In order to detect clear differences in soluble protein expression, three domain types viz. ATS, CIDR1α and DBL1α with a fairly wide range of solubility probabilities were selected for expression in *E. coli*. Additionally, the latter two domain types have been implicated in various cellular interactions and are therefore subject to regular attempts to obtain the same in a soluble form. A fairly simple and robust technique of colony blot filtration was employed to document soluble protein expression. This technique relies on the induction and subsequent lysis of *E. coli *colonies on a filter membrane. The soluble proteins diffuse through the filter membrane and are captured on to a nitrocellulose membrane placed under the filter membrane. The cellular debris and inclusion bodies are retained on the filter membrane or subsequently removed during the washing steps. Figure 4 clearly shows variation in the level of recombinant expression as soluble protein for these three PfEMP1 domain types. Detection with antibodies to fusion tags shows spots indicating the presence of soluble protein on the blots, which correspond to the number of colonies expressing the cloned domain. Extremely low signals from the blot harboring colonies expressing the DBL1α PF13 0003 domain, confirms the computational solubility assignment for this particular domain (6.9%). On the contrary, the intensity of signals from colonies expressing an equally long but a different domain type (CIDR1α) is intense, indicating a higher yield of soluble protein. A similar outcome was predicted by computational analysis, which assigned this particular domain a solubility probability of 79%. As predicted, the yield of soluble protein was highest on expression of an ATS domain, as evident from the intensity of the dots on the colony blot.

**Figure 3:**
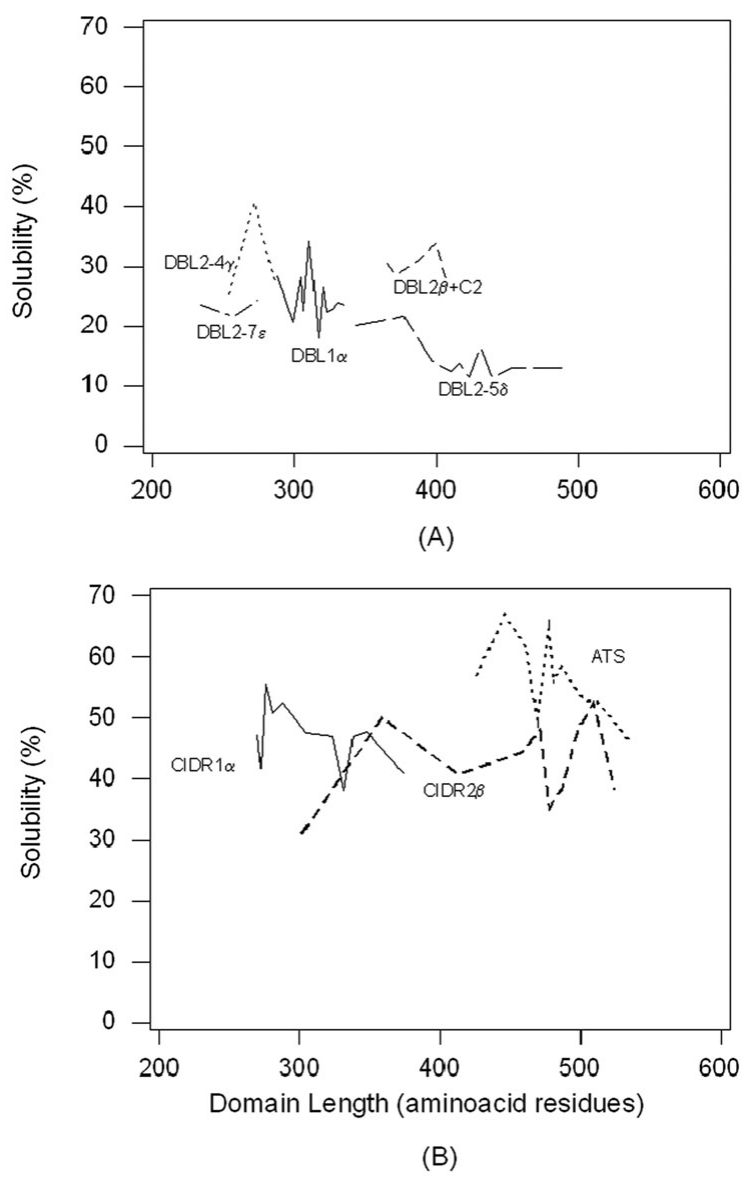
**Computational predictions (%) for soluble expression as a function of the length of the domain**. Representatives of each domain type were sorted out in the increasing order of their length. Means of domain lengths of blocks of 5 domains were plotted against the mean solubility predictions (%) for the corresponding five domains in the block. **A) **DBL domains are indicated as follows: DBL1α (solid line), DBL2β + C2 (dashed line), DBL2-4γ (dotted line), DBL2-5δ (long-dashed line), DBL2-7ε (combined dash-dotted line). **B) **ATS domains are indicated by a dotted line, CIDRα by a solid line and CIDR2β domains by a dashed line.

**Figure 4:**
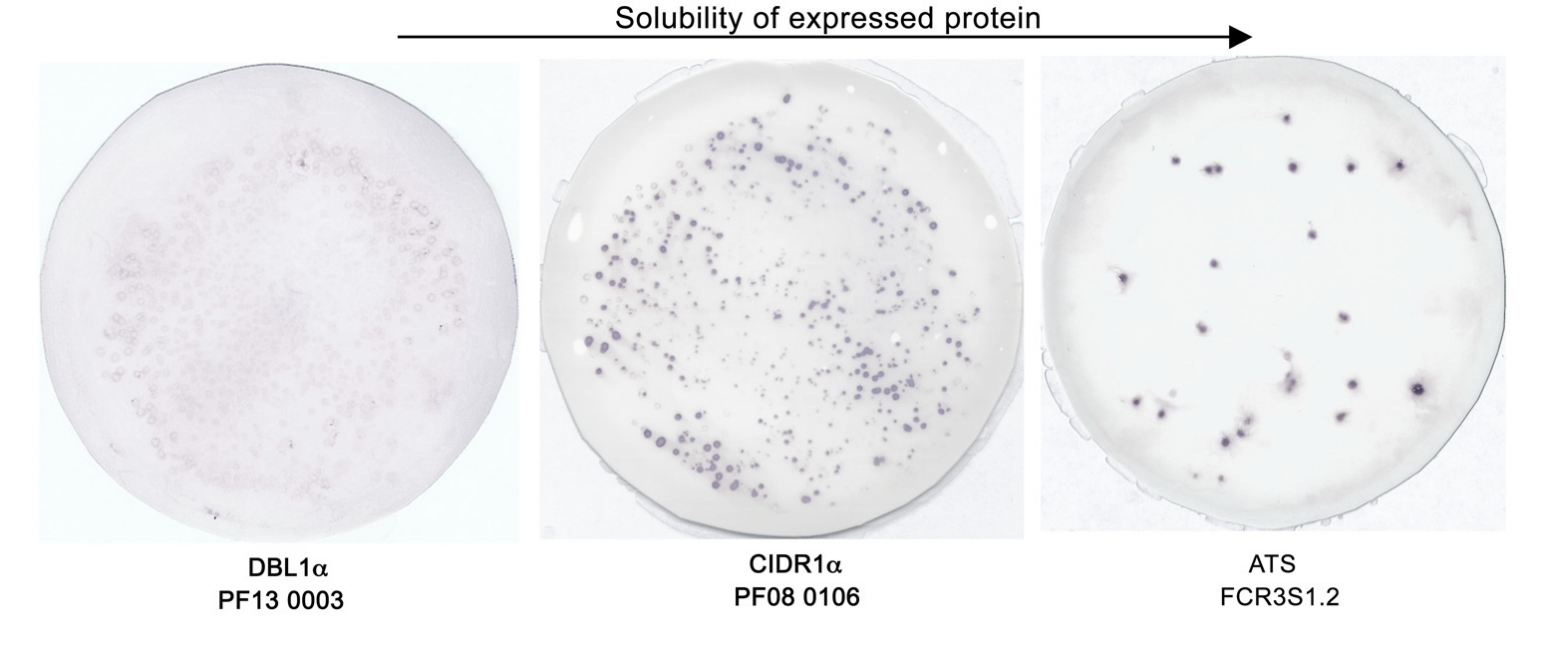
**Colony Filtration Blots for identification of soluble protein expression in *E. coli***. *E. coli *colonies expressing domains ATS, CIDR1α and DBL1α were induced and lysed on a membrane filter. Soluble protein from each cell within the colony diffuses through the filter and is captured on a nitrocellulose membrane placed under the membrane filter [13]. Detection with antibody reagents directed against the fusion tag, shows spots indicating the presence of soluble protein. Intensity of the spot corresponds to the yield of soluble protein. The arrow shows the increasing expression of soluble protein for the PfEMP1 domain types compared.

No correlation between the length of the domain and the probability of expression in a soluble form (Figure 1) was observed. In fact, the largest of the domains in the dataset, ATS and CIDR2β, with mean ± SD domain lengths of 482 ± 29 and 454 ± 72 amino acid residues respectively, were predicted to give higher soluble protein on expression in *E. coli*. DBL2-7ε domains, although being smallest (254 ± 19 amino acid residues) in the whole dataset, were however predicted to be confined to insoluble states on recombinant expression. The fates of DBL1α and CIDR1α domains, with similar mean domain lengths of 314 ± 13 and 310 ± 38 amino acid residues respectively, were predicted to dimensionally opposite states on recombinant expression. While CIDR1α domains were predicted to give soluble protein on *E. coli *expression, the opposite was true for DBL1α domains. Further, as Figure 4 confirms, DBL1α and CIDR1α domains of identical lengths (318 amino acid residues), gave different intensity of spots when probed for soluble protein expression in *E. coli*.

An attempt was made to study the variation in predictions for expression in soluble form over the length of each domain type (Figure 3A and 3B). Sequences of each domain type were sorted in the increasing order of their lengths. Means of domain lengths of blocks of five domains were plotted against the mean solubility predictions (%) for the corresponding five domains in the block. As expected, no unambiguous relationships between predictions for soluble expression and increase in size of the domain could be observed. This underscores the fact that sequence *per se *and not its length is the primary determinant of the fate of a domain to be expressed in a soluble or insoluble state. Avoiding stretches in domains predicted to drag the protein to insoluble states could thus be excluded when attempting recombinant expression in *E. coli *[14].

Expression of heterologous genes especially of malarial origin in *E. coli*, often results in the accumulation of highly pure recombinant protein in a totally or partially unfolded conformation as insoluble aggregates commonly referred to as inclusion bodies [15]. The major determinants of expression for many proteins are the thermodynamic and kinetic properties of an intermediate, rather than by the solubility and stability of the final state. Many turn-forming residues slow down protein folding, resulting in a high concentration of folding intermediates incompatible with folding rates resulting in aggregation of the protein. Solubility has also been correlated inversely to the hydrophobic nature of the protein [16]. The total charge of the aggregation-prone state of a protein strongly influences its propensity to aggregate. The propensity of charged residues as "structural gatekeepers" against aggregation does not, however, appear to be based only on the ability of these residues to interrupt contiguous stretches of hydrophobic residues, but also their ability to generate electrostatic repulsions between protein molecules. The higher the net charge, the higher the repulsion between proteins and hence lower the probability for aggregation. Appropriate interactions involving key residues i.e. cysteine also ensures that a newly synthesized chain of amino acids transforms into a correctly folded protein. But the mechanisms by which these residues affect the solubility status of an expressed protein remain to be understood.

Besides, the primary sequence of a protein, a multitude of external parameters such as culture media composition, growth temperature, production rate as a function of plasmid copy, promoter strength, codon usage and mRNA stability, all have a decisive influence on the expression of recombinant proteins. Even if the solubility algorithms predict a particular sequence to be confined to an insoluble state, alterations in these parameters can have an overriding effect on the final outcome [1]. Changing expression hosts can, however, be the preferred alternative to the time consuming and often uncertain option of optimizing physical parameters to obtain soluble protein. *In vitro *refolding procedures, although specific for each protein, are another useful option to obtain native protein in a non-high throughput setting [17].

As illustrated in the case of *P. falciparum *apical membrane antigen 1, recombinant proteins derived from *E. coli *or alternate expression hosts are functionally and immunologically equivalent [18]. Glycosylation patterns introduced on proteins expressed in non – *E. coli *expression hosts often impede crystallographic studies. Substantial evidence is beginning to surface regarding the functionality and immunological equivalence of merozoite surface protein purified and refolded from bacterial inclusion bodies [19]. Additionally, the fact that a significant proportion of vaccine candidates in the developmental pipeline have been expressed in *E. coli *provides ample proof of the unfailing enthusiasm for recombinant expression in *E. coli*.

Conversion of sequence data into biological reagents, namely proteins for experimental validation and characterization pose a significant challenge. Studying the primary protein sequence can, however, lend important inferences regarding the correlations between solubility and successful expression. A database assembling the successes and failures of recombinant expression of malarial genes in *E. coli *can further complement the accuracy of algorithms predicting soluble expression. Consequently, resources and costs can be channeled appropriately to expedite the delivery of a much-needed vaccine against *P. falciparum *malaria.

## Conclusion

Recombinant expression of malarial genes in *E. coli *is well recognized for its unpredictability. Screening of PfEMP1 domains for solubility traits for recombinant expression in *E. coli *allows prediction of the final outcome and can be applied as a valid tool for pursuing functional and structural studies on these domains.

## Authors' contributions

SA conceived the study, did computational analysis, performed validation of solubility predictions and drafted the primary manuscript. SPA provided valuable input on the biochemical aspects of the study. MW and QC participated in the analysis of data and contributed to the writing of the manuscript.
